# Accelerating river blindness elimination by supplementing MDA with a vegetation “slash and clear” vector control strategy: a data-driven modeling analysis

**DOI:** 10.1038/s41598-019-51835-0

**Published:** 2019-10-24

**Authors:** Morgan E. Smith, Shakir Bilal, Thomson L. Lakwo, Peace Habomugisha, Edridah Tukahebwa, Edson Byamukama, Moses N. Katabarwa, Frank O. Richards, Eddie W. Cupp, Thomas R. Unnasch, Edwin Michael

**Affiliations:** 1 Department of Biological Sciences, University of Notre Dame, Notre Dame, IN USA; 2 Vector Control Division, Ministry of Health, Kampala, Uganda; 3 The Carter Center, Uganda office, Kampala, Uganda; 4 Emory University and The Carter Center, Atlanta, GA USA; 5 Department of Entomology and Plant Pathology, Auburn University, Auburn, AL USA; 6 Department of Global Health, College of Public Health, University of South Florida, Tampa, FL USA

**Keywords:** Parasitic infection, Population dynamics, Applied mathematics

## Abstract

Attention is increasingly focusing on how best to accelerate progress toward meeting the WHO’s 2030 goals for neglected tropical diseases (NTDs). For river blindness, a major NTD targeted for elimination, there is a long history of using vector control to suppress transmission, but traditional larvicide-based approaches are limited in their utility. One innovative and sustainable approach, “slash and clear”, involves clearing vegetation from breeding areas, and recent field trials indicate that this technique very effectively reduces the biting density of *Simulium damnosum s.s*. In this study, we use a Bayesian data-driven mathematical modeling approach to investigate the potential impact of this intervention on human onchocerciasis infection. We developed a novel “slash and clear” model describing the effect of the intervention on seasonal black fly biting rates and coupled this with our population dynamics model of *Onchocerca volvulus* transmission. Our results indicate that supplementing annual drug treatments with “slash and clear” can significantly accelerate the achievement of onchocerciasis elimination. The efficacy of the intervention is not very sensitive to the timing of implementation, and the impact is meaningful even if vegetation is cleared only once per year. As such, this community-driven technique will represent an important option for achieving and sustaining *O. volvulus* elimination.

## Introduction

Large-scale initiatives aiming to control and eliminate neglected tropical diseases (NTDs) have made significant progress in treating at-risk populations and reducing the transmission and burden of these communicable diseases^1^. As NTD programmes achieve disease-specific targets set by the World Health Organization (WHO) Roadmap and enter the endgame phase of elimination, priorities will need to shift to adapt to changing transmission dynamics^2,3^. Novel approaches will be required to sustain elimination in the long term in the face of new infection patterns, emerging drug resistance, and socio-political challenges that are associated with the endgame^3–5^. Identifying the best course of action is not trivial, as complex socio-ecological systems are characterized by significant uncertainties, trade-offs between human action and ecological responses, and nonlinear effects that make elimination unpredictable and difficult to achieve^6^. Furthermore, attention is also increasingly focused on how best to accelerate progress toward meeting the WHO goals of eradication, elimination, or control of the major NTDs by 2030^1^. Recent work highlights that the development of intensified and diversified strategies are needed to accelerate the achievement of these targets^1,5,6^.

Vector-borne diseases are responsible for a large proportion of the global communicable disease burden^1^. Vector control (VC) is recognized by the WHO as a major tool to prevent the transmission of vector-borne NTDs, but is generally underused. There is, however, a long history of using VC in control and elimination efforts, particularly for onchocerciasis^7^. VC through the application of larvicides was the primary strategy of the Onchocerciasis Control Programme in West Africa^8–10^. The Ugandan experience with VC, when used in conjunction with twice per year treatment with ivermectin (IVM), is also rich and impressive; transmission for example has been interrupted in 15 of 17 endemic foci using this integrated approach^11–19^. However, the implementation of larvicide treatments can be labor-intensive and cost-prohibitive^20,21^. Insecticide resistance is also a major concern that threatens the long term success of pesticide-based interventions^1^. Innovative and sustainable VC interventions to support endgame elimination activities are thus critically needed.

Recently, building on this tradition of using VC for facilitating onchocerciasis elimination and drawing on similar approaches used previously in Sudan^22^ and Kenya^23^, Jacob *et al*.^24^ reported on the impact of vegetation clearing to reduce the biting density of *Simulium damnosum sensu stricto*, an important vector in the Madi mid-North focus in northern Uganda. The Jacob *et al*.^24^ “slash and clear” trials involved recruiting young men from villages in northern Uganda and training them to cut trailing vegetation from rivers. The vegetation was thrown on the riverbanks to dry, thereby killing any attached black fly larvae. Biting rates in the intervention villages were quickly reduced by up to 89–99%^24^. Lesser but still significant reductions were observed up to 120 days after the intervention was complete. The intervention was well-accepted by community members who were motivated to reduce the biting of black flies, and it was inexpensive requiring only basic materials already available in the villages. These results of the initial trials suggest that the “slash and clear” technique is highly promising for interrupting transmission in a cost-effective and sustainable manner.

The impact of “slash and clear” on onchocerciasis transmission and community infection when combined with mass drug administration (MDA) is, however, unknown. Preventive chemotherapy with ivermectin is the primary tool used against *Onchocerca volvulus* transmission, so understanding the value of clearing vegetation in combination with MDA is crucial if this tool is to support elimination programmes. Mathematical models offer a mechanism for investigating this key question in the absence of empirical information. Furthermore, forecasts made by models present decision makers with new information not otherwise available as data provides only retrospective insight. Thus, while the Jacob *et al*.^24^ trials present critical observations about the impact of “slash and clear” on vector biting rates, short-term data on their own cannot anticipate changes as a result of future management actions or ecological shifts, especially for biologically and socially complex systems^25^. By combining data with forecast models, the trial observations can also in addition be extrapolated to diverse settings and future scenarios. Moreover, forecast models have a unique ability to account for uncertainties in initial conditions, transmission drivers, and parameters to allow understanding of the full range of possible outcomes in different local settings^25^.

In this study, we developed a mathematical model of the “slash and clear” intervention to investigate the potential for this VC strategy to enhance efforts to accelerate progress toward onchocerciasis elimination. We present a novel mechanistic “slash and clear” model developed using the data from the field trials presented in Jacob *et al*.^24^. As part of this development, we also introduce a new seasonal black fly biting rate model. We couple this intervention model with our population dynamics model of onchocerciasis transmission^26^ to evaluate the benefit of supplementing MDA with “slash and clear” VC for accelerating transmission interruption. Specifically, our Bayesian data-driven modeling approach involves modeling baseline infection data from onchocerciasis endemic villages and then using the locally calibrated models to forecast the impacts of different “slash and clear” intervention scenarios. Our forecasts suggest that “slash and clear”, when used in conjunction with IVM MDA, can significantly accelerate the achievement of onchocerciasis elimination across endemic settings.

## Results

### Seasonal biting rate and “slash and clear” model

The “slash and clear” intervention involves removing vegetation from breeding sites (fast flowing, well oxygenated, sediment free water) along rivers to disrupt black fly breeding activities. Black fly breeding, and consequently biting intensity, typically varies throughout the year due to environmental changes, indicating the importance of considering the timing of the intervention in relation to these background seasonal patterns. Here, we developed a novel data-driven black fly biting rate model that considers seasonal fluctuations to account for this variation when simulating the “slash and clear” vector control intervention investigated in this study. The time-dependent biting rate was modelled by identifying the mechanistic relationship between the monthly biting rate (MBR) and expected monthly rainfall (see Methods). The model was calibrated to the observations of both variables reported for the geographical area of interest in Jacob *et al*.^24^.

**Figure 1:**
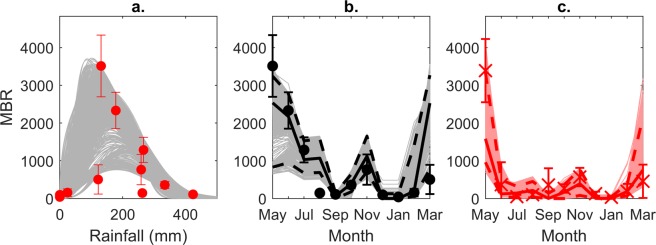
Model-predicted monthly biting rate as a function of rainfall in control and intervention sites. (**a**) The model predictions (gray curves) represent the MBR in the presence of seasonal rainfall fluctuations but in the absence of the “slash and clear” intervention. The ensemble of models together captured 91% of the observed MBR vs. rainfall data (red points with 95% confidence interval error bars) from the control sites for May 2017 – March 2018^24^. (**b**) Given the observed rainfall, the ensemble of models captured 91% of the data points from the control sites. (**c**) Given the observed rainfall, the ensemble of models captured 100% of the data points from the intervention sites.

Given the observed monthly rainfall during the long term trial^24^, our new double Weibull biting rate model captured 91% of the MBRs observed in the control sites, thereby supporting the process equation used to describe MBR as a function of rainfall (Fig. 1a). The model estimates of MBR in both the control and intervention (i.e. clearing vegetation in May) sites during the trial are shown in Fig. 1b,c. The ability of our exponential decay “slash and clear” intervention model (see Methods) to reproduce the observations is confirmed by these results with the ensemble of model predictions capturing 100% of the intervention data. The posterior median and 95% confidence intervals of the “slash and clear” efficacy (*η*) and efficacy decay rate (*Λ*) were *η* = 0.84 (0.75,0.95) and *Λ* = 0.16 (0.05, 0.39) after fitting the model to MBR data from the three intervention sites. These results indicate that the biting rate is immediately reduced by 84% following vegetation clearance and that the half-life of the intervention is ln(2)/*Λ* = 4.3 months. This means that the intervention can still remain 50% effective after about 4 months.

In our scenario modeling, we used a non-stationary cosinor model for estimating the seasonal rainfall pattern in northern Uganda to predict MBR as a function of monthly rainfall. The use of the cosinor model allowed us to consider uncertainty and non-stationarity in rainfall patterns from year to year as opposed to relying solely on the observed 2017–2018 rainfall. The model was fitted to the observed rainfall data for May 2017 - April 2018 in Gulu, Uganda^24^, and the fit is shown in Supplementary Fig. S1.

### Transmission model fits to site-specific baseline prevalence

Our Bayesian Melding (BM) modelling framework relies on data assimilation to discover local transmission models. Supplementary Fig. S2 shows the BM fits of our *O. volvulus* transmission model to the age microfilariae (mf) prevalence data in each of the four study sites (see Methods for a description of the model and study sites). Because age-stratified mf prevalence data were not available, two infection patterns (plateau and convex) were estimated from the observed overall prevalence, and each were equally considered in the model fitting procedure (see Supplementary Information (SI)). These results overall highlight the flexibility of the data-model assimilation approach for both overcoming gaps in data and for accurately differentiating between the prevailing transmission dynamics across endemic settings.

### mf and ATP breakpoint calculations

The model calibration procedure produced a selection of *n* = 500 best-fitting posterior parameter vectors from the initially drawn *N* = 200,000 samples (see Methods for the detailed procedure). For each of the selected parameter vectors, we calculated the mf prevalence and annual transmission potential (ATP) breakpoints at both the model-estimated annual biting rate (ABR) and threshold biting rate (TBR), resulting in a site-specific distribution for each of these variables. Using the empirical inverse cumulative distribution function of the ensemble of breakpoint values and exceedance probability calculations, we identified the breakpoints representing 95% elimination probability for each site (Table 1). These values served as the target endpoints for intervention programmes in subsequent simulations and analyses. Kruskal-Wallis tests indicated that the breakpoint distributions at ABR and TBR statistically significantly varied between sites, suggesting the existence of spatial heterogeneity in transmission dynamics (p-values < 0.003). It is also important to note that the mf breakpoints at TBR are statistically significantly higher than at ABR when vector populations are unperturbed (p-values < 1e-4), emphasizing the valuable role that VC can play to raise the transmission thresholds and facilitate the achievement of elimination. Significantly, the ATP values estimated in this study (Table 1) also indicate that the relevant ATP threshold values in a site may be different from the global value of 20 ATP set by WHO. Thus, if VC is not implemented, it is clear that the ATP thresholds at ABR may be significantly lower than the globally-set value of 20 (Table 1). However, if VC is used, it can be seen that the relevant ATP thresholds estimated at TBR could range from 16–91 across sites, a result which again highlights the important role that VC can play in facilitating the elimination of a vector-borne parasitic disease

**Table 1:** Model-predicted threshold values for mf and ATP indicators.

Village	mf breakpoint (%)		ATP threshold	
	TBR	ABR	TBR	ABR
Palaure Pacunaci	0.716	0.085	91.5	1.8
Masaloa	0.989	0.124	44.6	2.9
Nyimanji	0.802	0.133	28.7	3.0
Olimbuni/Aroga	0.455	0.115	16.1	2.7

Threshold values represent 95% elimination probability at the modelled site-specific ABR and TBR.

### Impact of “slash and clear” on required durations of interventions for achieving parasite elimination

**Figure 2:**
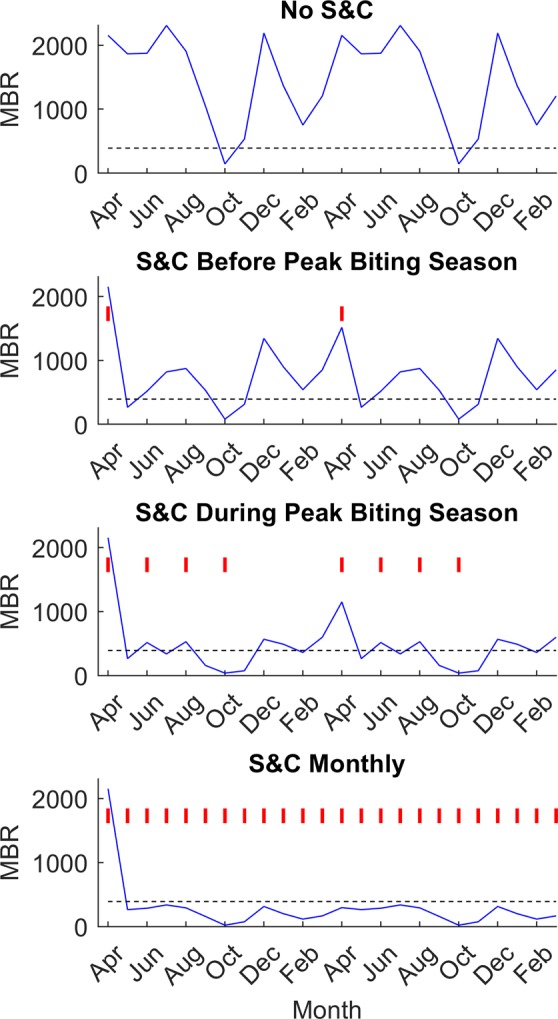
Impact of “slash and clear” on MBR for different intervention schedules in Masaloa, Uganda. Two years of implementing “slash and clear” is shown with vertical red lines indicating the months where vegetation was cleared. The blue line depicts the median MBR prediction throughout the intervention period and the horizontal black dashed line represents the median TBR for Masaloa.

**Figure 3:**
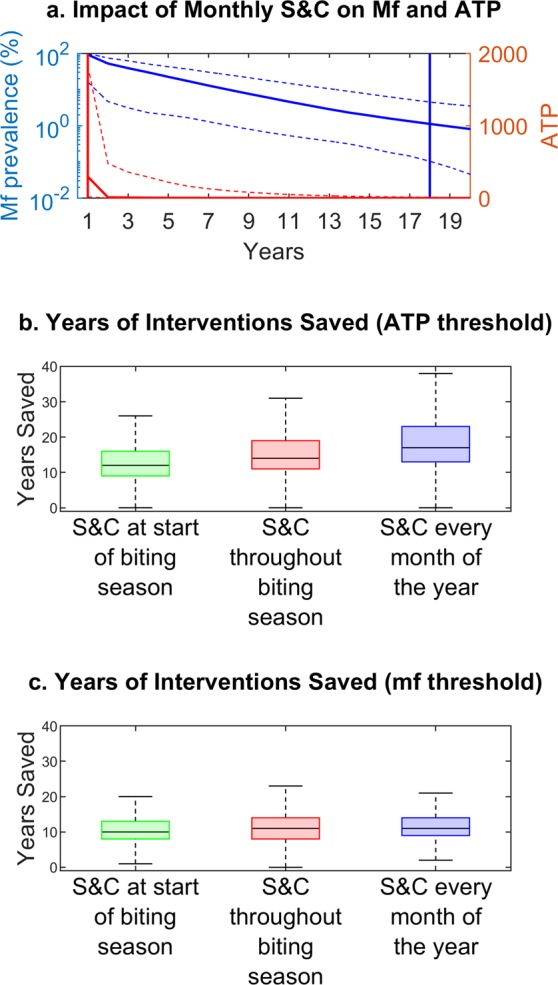
Impact of “slash and clear” on timelines to suppress or interrupt transmission. (**a**) Timelines to achieve the site-specific mf and ATP thresholds for Masaloa, Uganda when annual MDA is supplemented with monthly “slash and clear”. The blue curve shows the mf prevalence predictions over time (95% confidence bands shown by dashed lines) with the time required to reach the 95% elimination probability threshold given by a vertical blue line (18 years). The red line shows the ATP over time (95% confidence bands shown by dashed lines) with the time required to reach the 95% elimination probability threshold given by a vertical red line (1 year). (**b,c**) Years of interventions saved by supplementing annual MDA with “slash and clear” (S&C). Results for three different “slash and clear” schedules and two different elimination thresholds (modelled ATP (**b**) and mf (**c**) breakpoints) are shown. The results for all four study sites are pooled together. The whiskers correspond to 1.5 times the interquartile range.

We modelled several different “slash and clear” scenarios in combination with annual MDA to assess the optimal frequency and timing of the intervention and to evaluate the intervention’s potential to accelerate the achievement of onchocerciasis elimination. Three different scenarios were tested (see Methods): clearing vegetation (1) during the first month of the rainy season (here considered to be April), (2) once every two months during the rainy season (April – December), and (3) every month throughout the year. Figure 2 shows the predicted impact of these intervention schedules on MBRs compared to when no VC is used. For each site, timelines to reach the site-specific 95% elimination probability mf breakpoint and ATP threshold values were calculated for each scenario (Fig. 3, Table 2). Similar calculations were done for the WHO-defined threshold values (mf prevalence = 1%, ATP = 20^27,28^).

**Table 2:** Number of years of interventions required to reach mf and ATP transmission thresholds.

Village (baseline mf prevalence (%))	mf threshold				ATP threshold			
	No S&C	S&C before peak biting season	S&C during peak biting season	S&C monthly	No S&C	S&C before peak biting season	S&C during peak biting season	S&C monthly
**Model-predicted thresholds**								
Palaure Pacunaci (100)	34 (24–49)	26 (16–45)	25 (16–43)	24 (16–41)	28 (16–50)	10 (2–23)	8 (1–18)	4 (1–12)
Masaloa (76)	31 (19–49)	19 (11–33)	19 (10–31)	18 (10–29)	20 (10–34)	7 (1–17)	5 (1–14)	1 (1–9)
Nyimanji (58)	30 (18–47)	19 (10–34)	19 (10–33)	18 (10–32)	18 (8–33)	7 (1–18)	5 (1–14)	1 (1–9)
Olimbuni/Aroga (24)	28 (15–46)	20 (9–38)	19 (9–36)	19 (9–34)	17 (8–32)	8 (1–18)	5 (1–14)	1 (1–9)
**WHO thresholds**								
Palaure Pacunaci (100)	25 (15–45)	24 (15–41)	23 (15–40)	22 (14–37)	19 (9–45)	16 (7–32)	13 (4–26)	9 (1–19)
Masaloa (76)	20 (11–34)	19 (10–32)	19 (10–31)	18 (10–29)	13 (4–25)	10 (1–21)	8 (1–18)	1 (1–12)
Nyimanji (58)	19 (9–34)	18 (9–31)	17 (9–30)	17 (9–29)	11 (2–24)	8 (1–20)	6 (1–16)	1 (1–10)
Olimbuni/Aroga (24)	15 (5–30)	14 (5–28)	14 (5–26)	14 (5–26)	10 (1–22)	7 (1–17)	4 (1–13)	1 (1–8)

The number of years of required interventions is reported as the median prediction with its 95% confidence interval. All “slash and clear” scenarios are in combination with annual MDA at 80% population coverage. Results for both the model-predicted site-specific thresholds (representing 95% elimination probability) and the global WHO thresholds are shown.

The results of the scenario modeling suggest that the time required to reach the ATP threshold is directly proportional to the frequency at which vegetation is cleared and the baseline infection intensity. The most notable result was that if vegetation is cleared every month, then transmission suppression is predicted to be achievable in as little as one year (Table 2). Interestingly, there is a relatively small added benefit to clearing vegetation several times throughout the rainy compared to only during the first month of the rainy season. These additional efforts reduce the years of required interventions by only a couple of years compared to clearing vegetation only once per year. For all sites, there was a statistically significant difference between the predictions for each schedule (Kruskal-Wallis p-values < 1e-4).

Supplementing annual MDA with “slash and clear” significantly reduces the timelines to reach the site-specific mf breakpoints, with the impact of the different clearing frequencies found to be statistically significantly different (Kruskal-Wallis p-values < 1e-4). However, the differences between these schedules for crossing mf breakpoints are practically not very notable compared to the results pertaining to using the ATP threshold targets (Fig. 3). The addition of VC does not directly result in significant reductions of the community mf prevalence, but instead raises the mf breakpoint value making it easier to reach through MDA. Another point of interest is that the ATP threshold is predicted to be reached well before the mf breakpoint (Table 2).

### Uncertainty analysis

We modelled the various “slash and clear” scenarios in combination with biannual MDA to assess the sensitivity of the results to the annual MDA strategy. The major conclusions of this analysis are that (1) the predicted timelines to reach the mf and ATP thresholds are shorter if MDA is given biannually (Supplementary Table S1) compared to annually (Table 2), and (2) adding “slash and clear” will still have an important impact when combined with biannual MDA. Thus, under a strategy relying on drug treatment alone, biannual MDA can save 5–13 years of treatment compared to annual MDA, highlighting the strength of the added drug pressure. The savings are dependent on the choice of threshold and baseline endemicity of a site. However, despite the accelerated timelines using twice per year treatment, adding supplemental “slash and clear” VC can still save as many as 13 years of interventions compared to biannual MDA alone.

It is unlikely that rainfall patterns will remain constant in the long term future. Because the simulations consider time scales over which we cannot rely on stationarity, we performed a sensitivity analysis to evaluate how well the “slash and clear” intervention would work given unforeseeable shifts in seasonality. We tested scenarios where the rainfall patterns shift forward by 1, 3, and 6 months while keeping the intervention schedules the same. Results from this analysis suggest that there is no significant change in the impact of “slash and clear”, suggesting that the frequency of the intervention has a stronger effect than the timing even in the presence of uncertain future seasonal changes in rainfall and hence transmission (Supplementary Table S2).

## Discussion

The major result of this study that is immediately relevant for onchocerciasis elimination programmes is that supplementing annual MDA programmes with community-directed VC by clearing vegetation can significantly accelerate the achievement of parasite elimination in a sustainable manner. A recent field study in northern Uganda indicated that clearing vegetation using a community-driven approach can significantly reduce *Simulium damnosum s.s*. biting rates, but no empirical study has yet been done to test how this will affect infection rates in humans^24^. Here, we address this gap explicitly by using a forecasting model to link the observed changes in vector biting rates to the expected infection dynamics in the human population.

Our study has highlighted three major advantages offered by the addition of “slash and clear” VC for achieving rapid and sustained onchocerciasis elimination. First, the main result of importance for onchocerciasis elimination programmes is that supplementing MDA with vegetation clearing activities can significantly accelerate the average timelines to achieve elimination targets (Table 2). The implementation of “slash and clear” can potentially save, on average, more than 10 years of interventions compared to relying on annual MDA alone if mf thresholds are used as elimination targets, and, notably, the savings could increase to more than 20 years if the corresponding ATP thresholds are used (Table 2). These savings were also apparent in the case of biannual MDA, although to a lesser extent. Other studies have also shown this value of adding VC to MDA, and together with the present results, it is increasingly clear that relying on MDA alone may not bring about onchocerciasis elimination in all settings^29,30^. Indeed, field evidence supports this modelling result, demonstrating that the use of annual MDA alone in many areas has not led to achievement of targets even after 19 years of applying the intervention^29,30^, while VC alone and VC together with MDA have played key roles in onchocerciasis elimination efforts in Uganda and elsewhere^11,14–16,31^. These results strongly indicate that diversifying options, such as including “slash and clear” VC into MDA programmes, will be required if *S. damnosum* associated onchocerciasis is to be most effectively eliminated from endemic locations.

Second, an exciting result of this study is that, because of the lasting effects of this intervention (remaining at least 50% effective for 4 months), significant reductions in biting rates can be achieved by clearing vegetation before and/or during the biting season, and that year-round intervention is not required (Table 2 and Fig. 2). While monthly clearing will very quickly reduce transmission below the model-predicted ATP thresholds (potentially within 1 year of interventions), performing “slash and clear” just once per year is enough to save up to 18 years of interventions compared to relying on annual MDA alone in a hyper-endemic setting like Palaure Pacunaci (Table 2 and Fig. 3). Choosing to undertake annual or seasonal vegetation clearance if the community does not have the capacity for monthly intervention will still save many years of interventions compared to using annual MDA alone even if optimal coverages are achieved (here simulated at 80%). As expected, fewer years of interventions were saved in settings with lower pre-control prevalence. This trend remained true even if seasonal patterns were to shift and the vegetation was cleared annually at a suboptimal time with respect to the biting season (Supplementary Table S2).

Finally, “slash and clear” is an alternative form of VC capable of alleviating challenges associated with MDA^5,21,32,33^. While the value of VC for eliminating onchocerciasis is well-established, the cost of implementing traditional larvicide-based approaches, the potential for larvicide resistance, and the amount of preparatory work required to implement this intervention are of concern^1,20^. In line with the growing trend of integrating community participation in VC efforts^34–37^, “slash and clear” can be considered an effective community-directed intervention in that community members are trained and are responsible for clearing the vegetation. Jacob *et al*.^24^ reports that communities were motivated to be involved because the biting of the black flies is very bothersome, suggesting that “slash and clear” would have sustained support by local populations. Furthermore, there are no costly materials required, removing the financial burden associated with larvicide treatments. Because this intervention involves environmental management as opposed to chemical solutions, the emergence of resistance would be a lesser threat to elimination, further improving the sustainability of the approach^38–40^.

Another notable feature of our study is the evaluation of two different transmission indicators, mf prevalence and ATP. Our findings show that ATP thresholds are reached markedly earlier than mf breakpoints (Table 2 and Fig. 3), suggesting that, in locations where vector migration is not a concern, targets based on indicators in the vector (ATP) are significantly more sensitive for detecting the eventual interruption of transmission than the corresponding indicators in the human (mf). However, as noted in Methods, in settings where the in-migration of black flies is likely, MDA will still be important for reducing the intensity of the remaining mf infections in order to achieve the permanent reduction of transmission^27^; here, adding “slash and clear” to continuing MDA interventions will still significantly reduce the number of years required for this extended drug intervention (Table 2). Furthermore, in these locations, the addition of VC into MDA programmes can serve to reduce the risk of recrudescence by making the system more resistant to the re-initiation of infections essentially by driving the prevailing ABR toward its local TBR, which in turn raises the mf breakpoint/emergence values^41^. This effect, coupled by the long-lasting impact of the present VC method against reestablishement of the fly population, suggests that introducing community-driven “slash and clear” VC in *S. damnosum* transmission areas nearing elimination targets may represent an important endgame strategy for ensuring onchocerciasis elimination. Given the rising concern about the impact of cross-border introduction of infection^33^, implementing “slash and clear” in a border region that has successfully interrupted transmission could also act as an effective strategy to safeguard the gains made. Field trials to evaluate these predicted impacts of the “slash and clear” VC are required to attest to the real-world usefulness of this approach as an onchocerciasis endgame/cross-border strategy.

Mf is a poor indicator for other reasons as well like low diagnostic sensitivity, especially in low prevalence settings, and community disapproval of the skin snip procedure, resulting in a general movement away from the use of mf as an indicator and toward the application of vector and serology-based indicators for evaluating onchocerciasis elimination^32,42,43^. However, identifying reliable vector and serology thresholds is still a key issue that needs to be resolved^43^. The vector thresholds recommended by WHO was initially set at a value of 20 ATP per site^27^. Our results on ATP threshold values (Table 1), however, have provided three important insights regarding the applicable vector threshold values in a site. First, as in the case of the corresponding mf breakpoint values, vector-based elimination thresholds (here ATP) will vary significantly between sites owing to variations in local transmission conditions. Second, the relevant ATP threshold values may be different from the global value of 20 set by WHO. Third, if VC is not implemented, the ATP thresholds at ABR may be significantly lower than the globally-set value of 20 but these will increase to 16–91 across sites if VC is used (Table 1). These are important results, and highlight: (1) that these thresholds are not spatially stationary but are properties of heterogeneous transmission dynamics, and (2) that currently set targets require revaluation.

This study emphasizes the valuable role that forecasting models can play in programme design and decision-making. While the field data reported from the “slash and clear” trials showed great promise for reducing *O. volvulus* transmission^24^, it is clear that such data alone may not provide insight into infection dynamics in humans nor can it be relied upon to anticipate the impact of the intervention in other settings or in the future. This highlights some of the challenges associated with using data alone for decision-making in parasite elimination. Thus, while data can provide critical evidence, it is important to note, firstly, that data on one state variable alone cannot predict the behavior of the overall transmission system and, in particular, does not guarantee the detection of change in related but separate parts of the system^25^. Combining data with forecasting models may, however, allow us to clearly define what we know, extrapolate this information to other settings, and reliably predict the outcome^25^. Furthermore, the use of data-assimilated models provides a framework for explicitly considering uncertainties in data, initial conditions, and parameters^25^. Here, we used a Bayesian Melding framework for expressing initial uncertainties in pre-control ABR (a key system driver) and other model parameters, which are updated or localized for a site based on mf infection data. This allows us to discover models with associated uncertainties for a given site which capture the properties of local dynamics better, and thus to make more reliable site-specific future forecasts of intervention outcomes^25^. Note that such data-model assimilation is flexible and can enable models to be easily updated as new data are collected, which can be a useful procedure for further reducing forecast uncertainty and thus supporting more reliable decision-making^44^.

A limitation to our study is that the modelled site-specific breakpoints and the WHO-proposed thresholds for onchocerciasis have not yet been sufficiently validated. Our modeling results, in agreement with others, indicate that breakpoints vary by endemicity and are lower than the operational thresholds currently used by global programmes (Table 1)^29,32,45^. Undoubtedly, this indicates that there is an urgent need for reevaluating and confirming the criteria used for determining whether transmission has been interrupted^43,45^. However, note that the key conclusion of our study, viz. that adding “slash and clear” VC to MDA will significantly reduce timelines to eliminate onchocerciasis transmission compared to using MDA alone, will not change but the actual timelines predicted would (compare the durations of interventions needed for meeting the model-presicted thresholds versus the WHO threshold given in Table 2). Future refinements of our model are also needed with respect to modeling seasonality and serological indicators. In this first development, we modelled the MBR as a function of monthly rainfall to capture seasonality and predict the impact of future shifts in seasonal patterns. This model was based on one year of rainfall data and should be updated with future and possibly historical rainfall data. Other relevant environmental factors that impact biting rate could also be incorporated, such as temperature^46^. Additionally, as the WHO has adopted serology indicators, it will be important to extend our model to consider this new indicator. Finally, we have modelled the efficacy of the vegetation “slash and clear” approach based on impact on MBR from only three experimental field sites. As the “slash and clear” efficacy parameters may depend on local vegetation and other riverine features, further field studies of the impact of this and indeed other approaches for reducing vector populations and hindering their regrowth based on vegetation clearance should be carried out in relevant settings to provide more reliable estimates of efficacy, and to determine how it may vary between diverse ecologies.

In summary, our data-driven modeling study predicts that clearing vegetation as a form of VC can significantly accelerate the achievement of elimination of *S. damnosum*-transmitted onchocerciasis, regardless of the timing and frequency of implementation. These results also highlight the value of supplementing annual MDA with VC, a conclusion that is applicable to many vector-borne NTDs. VC, especially implemented via a community-directed approach, such as embodied by the “slash and clear” technique studied here, may also overcome the challenges of chemical-based VC delivered using public infrastructures, and thus may constitute a more sustainable approach to deploying long-term VC. Further consideration of the optimal indicator and means of measurement to detect the interruption of vector-borne macroparasitic disease transmission is required. Our results suggest that measuring transmission status based on vector-related targets is more sensitive than using measures of infection in humans, but the choice of endpoint targets will depend crucially on whether significant dispersal of vectors occurs between intervention sites. Finally, this study emphasizes the value of using data-assimilation models for forecasting the effects of parasite intervention strategies even in data-limited situations.

## Methods

### Modeling seasonal black fly biting rates as a function of rainfall

To model seasonal fluctuations in MBR, we first fitted a non-stationary cosinor model to monthly rainfall data applicable to our observation sites (obtained from the Gulu meteorological station in northern Uganda for May 2017 - April 2018)^24,47^. We chose to use rainfall as a proxy for seasonality because, apart from being a well-established driver variable underpinning black fly population dynamics, there were reliable rainfall data published alongside the biting rate data in Jacob *et al*.^24^ thus creating an opportunity for modeling the observed correlation between rainfall and biting rate in the local setting. We then used the fits to predict the amount of rainfall in mm with associated 95% confidence intervals for each month of the year. The non-stationary cosinor model captures annual seasonality in time series data using a sinusoid and is flexible enough to allow for changes in seasonality over time. In subsequent simulations, we therefore did not assume that the observed rainfall remained constant from year to year, but instead drew random samples from the predicted intervals for each month to serve as inputs to the MBR function. The MBR is related to rainfall through a double Weibull function:

$$MBR(m)=MB{R}_{M}(1-{e}^{-{(\frac{{R}_{t}(m)}{{R}_{L}})}^{{k}_{1}}}){e}^{-{(\frac{{R}_{t}(m)}{{R}_{U}})}^{{k}_{2}}}$$

where *MBR*_*M*_ is the maximum expected biting rate, *R*_*U*_ and *R*_*L*_ represent upper and lower rainfall thresholds above and below which fly biting is greatly reduced, and *k*_1_ and *k*_2_ are shape parameters. We calibrated this rainfall-dependent seasonal MBR model to monthly biting data from control sites (Supplementary Table S3) using Bayesian Melding with a pass/fail filter (see description of BM approach below)^48–51^. The prior parameter ranges for *R*_*L*_, *R*_*U*_, *k*_1_ and *k*_2_ are given in Supplementary Table S4. The pass/fail filter was based on the following acceptance criteria: the ensemble of models was considered acceptable if >85% of the observed data points were captured.

### Biting rate reduction due to “slash and clear”

Removing trailing vegetation by the “slash and clear” technique was shown in trials to have a strong and rapid impact on black fly biting rates^24^. The biting rate was reduced by 89–99% within one month post-intervention in the intervention sites compared to control sites, and the reduction was long lasting with biting density significantly reduced for up to 4 months^24^. To capture this immediate decline followed by a slow period of population regrowth, we model the effects of “slash and clear” as an intervention acting against the MBR whose efficacy declines over time according to an exponential decay function:

$$MBR(m,{t}_{s})=MB{R}_{M}(1-{e}^{-{(\frac{{R}_{t}(m)}{{R}_{L}})}^{{k}_{1}}}){e}^{-{(\frac{{R}_{t}(m)}{{R}_{U}})}^{{k}_{2}}}\eta (1-{e}^{-\Lambda {t}_{s}})$$

where *η* is the immediate percentage decrease in biting rate due to the removal of vegetation, *Λ* is the decay rate of the efficacy of the intervention at maintaining reduced biting rates, and *t*_*s*_ is the time since clearing the vegetation in months. Note that, in this formulation, *Λ* controls the rate of return of the black fly population following the intervention. We calibrated the seasonal MBR model with the effects of “slash and clear” to monthly biting data from the intervention sites (Supplementary Table S3)^48–51^. The prior parameter ranges for *η* and *Λ* are given in Supplementary Table S4. The pass/fail filter was based on the same acceptance criteria as above.

### Survey data

To simulate “slash and clear” interventions in this modeling study, we modelled four onchocerciasis endemic sites in northern and western Uganda that closely resemble the environment from the original trials described in Jacob *et al*.^24^. *Simulium damnosum s.s*. is responsible for transmission in these sites, and together they also represented a range of transmission conditions. Table 3 provides the baseline microfilariae (mf) prevalence survey data used to calibrate the model to local conditions^13,17,26^. Baseline surveys were carried out in 1993–1994 for all sites using standard skin snip protocols for diagnosing the presence of mf^13,17,26^.

**Table 3:** Ugandan onchocerciasis study sites.

Focus	Village	Baseline Prevalence (%)	Ref.
Madi mid-North	Palaure Pacunaci	100	^26^
Madi mid-North	Masaloa	76	^26^
Wadelai	Nyimanji	58	^17^
Obongi	Olimbuni/Aroga	24	^13^

### Onchocerciasis transmission model

The biting rate model developed here was coupled to a filarial parasite transmission model previously parameterized for onchocerciasis^26^. The state variables of this model vary over age (*a*) and/or time (*t*) and track changes in the average pre-patent (*P*(*a*,*t*)) and adult (*W*(*a*,*t*)) worm burden per human host, the average microfilariae intensity per human host (*M*(*a*,*t*)) the average number of infective L3 stage larvae per black fly (*L*), and a measure of immunity (*I*(*a*,*t*)) developed by human hosts against incoming L3 larvae. The state equations comprising this model are:

$$\begin{array}{c}\frac{\partial P}{\partial t}+\frac{\partial P}{\partial a}=\Phi {L}^{\ast }{F}_{1}(I(a,t)){F}_{2}({W}_{T}(a,t))-{\mu }_{w}P(a,t)-\Phi {L}^{\ast }{F}_{1}(I(a,t-\tau )){F}_{2}({W}_{T}(a,t-\tau ))\zeta \\ \frac{\partial W}{\partial t}+\frac{\partial W}{\partial a}=\Phi {L}^{\ast }{F}_{1}(I(a,t-\tau )){F}_{2}({W}_{T}(a,t-\tau ))\zeta -{\mu }_{w}W(a,t)\\ \frac{\partial M}{\partial t}+\frac{\partial M}{\partial a}={F}_{3}({W}_{T}(a,t))-\gamma M(a,t)\\ \frac{\partial I}{\partial t}+\frac{\partial I}{\partial a}={W}_{T}(a,t)-\delta I(a,t)\\ {L}^{\ast }={F}_{4}({W}_{T}(a,t))\end{array}$$

Each function, *F*_*x*_, denotes a functional form for which the specified state variable serves as an input along with other model parameters. Expanded forms of these functions are fully described in previous work^26^ and all parameter values and functional forms are detailed in Supplementary Tables S5 and S6. Note that some functions are dependent on the total worm load where *W*_*T*_ = *W(a,t)* + *P(a,t)*. Given the faster time scale of infection dynamics in the vector compared to the human host, we make a simplifying assumption that the density of infective stage larvae in the vector population reaches a dynamic equilibrium rapidly^52–56^, so the infective L3-stage larval density in the vector population is evaluated as an ordinary differential equation at equilibrium (denoted by L^*^).

### Bayesian Melding fitting of the model to the baseline data

Our computational approach is founded on a BM data assimilation framework^48,55–58^. Using the known ranges of the model parameter values, we defined uniform prior distributions for each parameter and randomly sampled with replacement from these distributions to generate *N* = 200,000 parameter vectors. The model was run with each of the *N* parameter vectors, which generated *N* outputs predicting mf prevalence by age. Because there was no ABR data for the study sites, this parameter was also inversely estimated during this step to identify plausible ABR values for the geographic location and endemic prevalence. The model outputs were compared against age-stratified mf prevalence data by calculating binomial log-likelihoods for each parameter vector. Note that the datasets used in this work did not include age-stratified mf prevalence data, so the age profiles were derived from the observed overall community prevalence according to the procedure presented in Smith *et al*.^51^. The derivation of the age prevalence structures for onchocerciasis is given in the SI. Next, a Sampling-Importance-Resampling algorithm was used to select *n* = 500 samples with replacement from the pool of *N* parameter vectors with probabilities proportional to their relative log-likelihood values. This step generated the *n* parameter vectors most likely to describe the data. The *n* resampled posterior parameter vectors were used to compute distributions of variables of interest from the fitted model (ex. age-prevalence curves, worm breakpoints and infection trajectories following treatments). Note that this approach means that none of the biological parameters of the model are fixed *a priori*, but rather the initial priors are updated by the mf data and are thus informed by the local transmission setting^26^.

### Model-based calculations of site-specific transmission thresholds

Using a numerical stability analysis approach, we calculated the TBR and the mf prevalence breakpoints for each of the best-fitting parameter vectors^55,56,58,59^. To calculate the TBR for each parameter vector, we progressively decrease the average number of black flies per human, *m*, from its original value to a threshold value below which the model always converges to zero mf prevalence. The product of the number of bites per fly per month, *β*, and this newly found *m* value is termed as the TBR. We similarly calculate the mf prevalence breakpoints. Given a particular biting rate (either the ABR or TBR), we estimate the minimum L^*^ below which the model predicts zero mf prevalence. The corresponding mf prevalence at this threshold L^*^ value is termed as the mf breakpoint. The distribution of mf breakpoints at a particular biting rate in a site can be described by an empirical inverse cumulative density function, which we used in conjunction with exceedance calculations^59^ to quantify the values of mf breakpoint prevalence thresholds reflecting various elimination probabilities. Here, we used the mf breakpoint corresponding to an elimination probability of 95% as the elimination target.

In this study, we also consider the impact of “slash and clear” on the ATP thresholds, as ATP is an important entomological indicator considered in intervention programmes against onchocerciasis. The ATP is calculated as the product of the ABR and the population averaged number of L3 larvae per black fly^27,60^, and the ATP threshold thought to indicate that local transmission is no longer sustainable has been fixed at 20 by WHO^27^. Here, we modelled the site-specific ATP threshold as the product of the biting rate and L^*^ thresholds calculated for a site as described above (at either ABR or TBR). When VC is implemented, the TBR is assumed to be the relevant biting rate and the corresponding L^*^ threshold is used in the ATP calculations. When VC is not used, the ATP threshold is calculated by multiplying the ABR and the corresponding L^*^ threshold.

Note, according to epidemiological theory, crossing below either of these thresholds would lead to the cessation of vector-borne disease transmission in a local setting^55,61,62^. However, given the relatively longer life span of adult worms, it will invariably take more time to achieve the mf threshold compared to the ATP threshold. Furthermore, in an open environment (i.e. where dispersal or migration of flies is significant), the persistence of mf despite stopping local transmission once ATP thresholds are crossed would pose a risk of reestablishment of transmission if MDA is stopped before mf breakpoints are reached. This risk of recrudescence of infection means, as per the WHO definitions, that achieving permanent transmission interruption in a local setting may require waiting until the mf thresholds are also met, while meeting the ATP thresholds can be considered as conditional interruption or suppression of transmission^27^.

### Modeling the effects of mass drug administration

In our simulations, we consider the complementary impact of “slash and clear” when added to a standard onchocerciasis MDA programme with IVM. The impact of MDA was modelled by assuming that the drug acts by instantaneously killing certain fractions of the pre-patent worm (*P*), patent (*W*) adult worm, and mf (*M*) populations. We denote these fractions as *ω* for worms and *ε* for mf. Because there lacks consensus on the efficacy of annual IVM against adult *Onchocerca volvulus* parasites, we allow *ω* to vary from 0.05 to 0.3^63–71^. The population sizes of worms and microfilariae after drug treatment are calculated by modifying the populations of each parasite stage immediately prior to the treatment:

$$\begin{array}{c}P(a,t+dt)=(1-\omega C)P(a,t)\\ W(a,t+dt)=(1-\omega C)W(a,t)\\ M(a,t+dt)=(1-\varepsilon C)M(a,t)\end{array}\}{\rm{at}}\,{\rm{time}}\,t={T}_{MDAi}$$

In the above equation, $dt$ represents a short time-period since the time-point ${T}_{MD{A}_{i}}$ when the *i*^*th*^ round of MDA was administered. The parameter *C* is the population level drug coverage. As IVM is generally also thought to act by suppressing the production of mf by worms surviving each MDA^65,69^, we modelled this sterilizing effect by introducing the parameter *δ*_*reduc*_:

$$\frac{\partial M}{\partial t}+\frac{\partial M}{\partial a}=(1-{\delta }_{reduc}C)s\alpha \varphi [W(a,t),k]W(a,t)-{\mu }_{2}M(a,t),\,{\rm{for}}\,{T}_{MDAi} < t\le {T}_{MDAi}+P$$

where $\alpha ^{\prime} =\alpha (1-{\delta }_{reduc}C)$ reflects the suppressed fecundity (over a period of *T*_*P*_ months since the *i*^*th*^ MDA) of adult worms that survive the administration of drugs at each MDA.

### Policy scenarios

Because the “slash and clear” intervention acts to reduce the black fly population and the background population dynamics are highly variable by season, we hypothesized that the timing of the intervention would be important. We considered several different intervention schedules to investigate the impact that timing and frequency of the intervention will have on its efficacy. For all of these scenarios, the rainy season was considered to run from April through November and the dry season from December through March, following the observations in Jacob *et al*.^24^. Simulations were started at the beginning of the rainy season in April. In the first scenario, we assume that it is known when the rainy season (corresponding to the biting season) is expected to occur and we implement “slash and clear” during the first month of the biting season. In reality, the timing of peak biting is somewhat unpredictable. Therefore, in the second scenario, we considered clearing vegetation every other month during the biting season. This schedule was based on the proposal by Jacob *et al*.^24^ to clear vegetation every two months. Finally, we considered a third scenario where “slash and clear” is implemented monthly throughout the year. We model these intervention scenarios in combination with a standard annual MDA programme at the WHO-recommended coverage of 80%. For each intervention scenario, the intervention timelines to suppress or interrupt transmission, marked by the achievement of either the ATP threshold or mf breakpoint, respectively, were calculated for both site-specific and global WHO thresholds and then compared to quantify the impact of the intervention.

### Uncertainty Analysis

Note that while the study sites chosen are located in Uganda, the annual MDA scenarios do not reflect the Ugandan onchocerciasis elimination strategy. In order to be generally applicable, we modelled a standard annual MDA intervention strategy to investigate the combined effect of IVM and “slash and clear”, but in practice the Ugandan policy is moving towards enhancing their elimination programmes by implementing biannual MDA^12^. To assess the sensitivity of the results to the annual MDA programme, we repeated the scenarios described above under a biannual MDA programme also at 80% coverage. Additionally, it is unlikely that seasonal patterns will remain constant from year to year. Therefore, as a sensitivity analysis, we repeated these simulations while shifting the rainfall patterns by 1, 3, and 6 months to assess how sensitive the results are to the timing of the intervention in relation to the biting season.

## Supplementary information

Supplementary Information

## Data Availability

All data generated or analyzed during this study are included in this published article and its Supplementary Information Files. Model code is available at https://github.com/EdwinMichaelLab/SlashAndClear.
