# Supplementary material for: Accelerating river blindness elimination by supplementing MDA with a vegetation “slash and clear” vector control strategy: a data-driven modeling analysis
Source: Sci Rep. 2019 Oct 24;9:15274. doi: 10.1038/s41598-019-51835-0 (PMC6813336; doi:10.1038/s41598-019-51835-0)
Supplement: Supplementary file 1 — Supplementary Information [file 41598_2019_51835_MOESM1_ESM.docx]

Supplementary Information

Accelerating river blindness elimination by supplementing MDA with a vegetation “slash and clear” vector control strategy: a data-driven modeling analysis

Morgan E. Smith, Shakir Bilal, Thomson L. Lakwo, Peace Habomugisha, Edridah Tukahebwa, Edson Byamukama, Moses N. Katabarwa, Frank O. Richards, Eddie W. Cupp, Thomas R. Unnasch, Edwin Michael


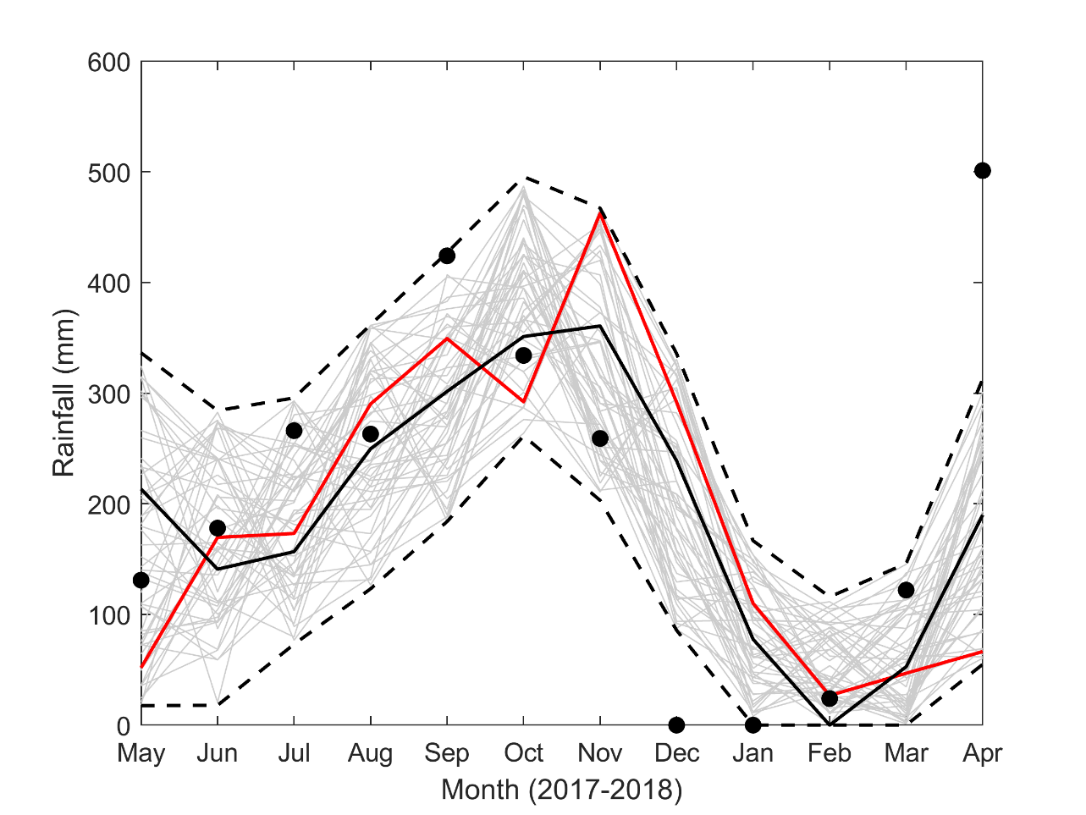


Supplementary Figure S1. Non-stationary cosinor model rainfall estimate (mean shown as solid black line, 95% confidence interval shown as dashed black lines) compared to observed rainfall data (black points) for the Gulu region, Uganda for May 2017-April 2018. Data was obtained from Jacob et al. ^1^. The gray lines show randomized rainfall patterns used for simulating interventions with one example highlighted in red. While the model does not capture the April data point, this observation is a suspected outlier compared to average rainfall trends (https://www.weather-atlas.com/en/uganda/gulu-climate).


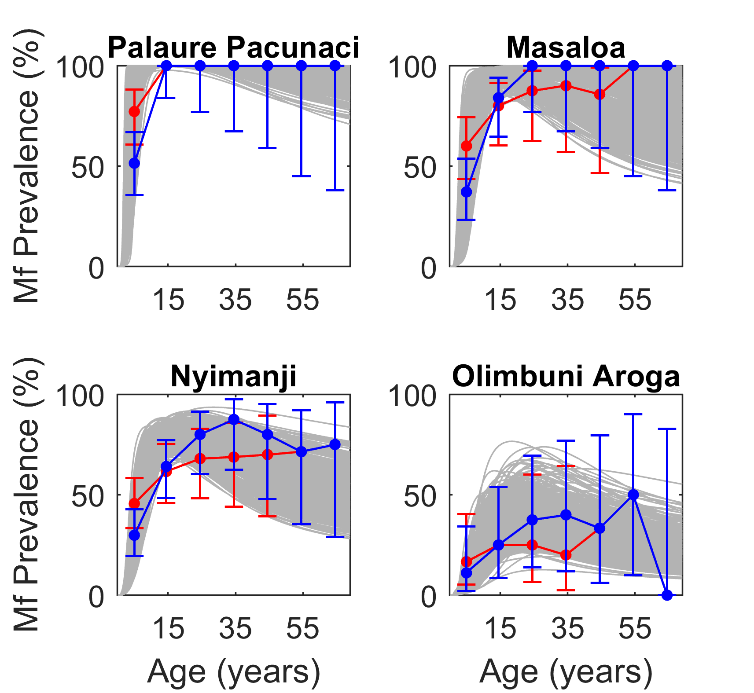


Supplementary Figure S2. Onchocerciasis model fits to constructed age prevalence data for four endemic sites in Uganda. The gray curves are the age prevalence patterns predicted by an ensemble of best-fitting models for the given site. Age-stratified data were derived from the observed overall community baseline prevalence for plateau (red) and convex (blue) patterns.

Supplementary Table S1. Number of years of interventions required to reach mf and ATP transmission thresholds using biannual MDA at 80% coverage.

| **Village**  **(baseline mf**  **prevalence (%))** | **mf threshold** | | | | **ATP threshold** | | | |
| --- | --- | --- | --- | --- | --- | --- | --- | --- |
|  | **No S&C** | **S&C before peak biting season** | **S&C during peak biting season** | **S&C**  **monthly** | **No S&C** | **S&C before peak biting season** | **S&C during peak biting season** | **S&C monthly** |
|  | **Model-predicted thresholds** | | | | | | | |
| Palaure Pacunaci (100) | 23 (14-44) | 15 (9-30) | 15 (9-30) | 15 (8-29) | 15 (8-30) | 5 (1-12) | 4 (1-10) | 2 (1-7) |
| Masaloa (76) | 19 (11-37) | 11 (5-21) | 11 (5-21) | 11 (5-21) | 11 (5-22) | 3 (1-9) | 2 (1-8) | 1 (1-4) |
| Nyimanji (58) | 18 (10-35) | 11 (5-23) | 11 (5-22) | 11 (5-22) | 10 (4-21) | 3 (1-10) | 2 (1-8) | 1 (1-4) |
| Olimbuni/Aroga (24) | 17 (9-34) | 11 (5-25) | 11 (5-25) | 11 (5-24) | 9 (4-19) | 4 (1-10) | 2 (1-8) | 1 (1-5) |
|  | **WHO thresholds** | | | | | | | |
| Palaure Pacunaci (100) | 14 (8-30) | 13 (7-28) | 13 (7-27) | 13 (7-26) | 10 (4-22) | 8 (3-18) | 7 (1-16) | 5 (1-12) |
| Masaloa (76) | 11 (4-22) | 11 (4-21) | 11 (4-21) | 11 (4-21) | 7 (2-15) | 5 (1-12) | 3 (1-10) | 1 (1-7) |
| Nyimanji (58) | 10 (4-21) | 10 (4-20) | 10 (4-20) | 10 (4-20) | 6 (1-13) | 4 (1-11) | 3 (1-9) | 1 (1-5) |
| Olimbuni/Aroga (24) | 8 (1-19) | 8 (1-18) | 8 (1-18) | 7 (1-18) | 5 (1-12) | 3 (1-9) | 1 (1-7) | 1 (1-4) |

Supplementary Table S2. Median (95% CI) number of years of annual MDA and VC required to reach transmission thresholds given shifts in seasonal patterns.

|  | **Village** | **mf threshold** | | | | **ATP threshold** | | | |
| --- | --- | --- | --- | --- | --- | --- | --- | --- | --- |
|  |  | **No S&C** | **S&C one**  **month before peak season** | **S&C during peak season** | **S&C monthly** | **No S&C** | **S&C one**  **month before peak season** | **S&C during peak season** | **S&C monthly** |
| **Model-predicted**  **site-specific**  **thresholds**  **(95% elimination probability)** | **shift by 1 month** | | | | | | | | |
|  | Palaure Pacunaci | 35 (24-49) | 25 (16-43) | 25 (16-43) | 24 (16-40) | 27 (15-50) | 10 (2-21) | 7 (1-17) | 4 (1-12) |
|  | Masaloa | 31 (19-48) | 19 (11-33) | 19 (10-31) | 18 (10-29) | 20 (11-33) | 7 (1-16) | 4 (1-13) | 1 (1-9) |
|  | Nyimanji | 30 (18-47) | 19 (10-34) | 19 (10-33) | 18 (10-32) | 18 (8-33) | 7 (1-17) | 4 (1-13) | 1 (1-8) |
|  | Olimbuni Aroga | 28 (16-47) | 20 (9-38) | 20 (9-35) | 19 (9-34) | 16 (8-31) | 7 (1-17) | 5 (1-14) | 1 (1-8) |
|  | **shift by 3 months** | | | | | | | | |
|  | Palaure Pacunaci | 35 (25-49) | 26 (16-44) | 25 (16-42) | 24 (16-41) | 28 (16-50) | 10 (2-24) | 8 (1-18) | 4 (1-12) |
|  | Masaloa | 31 (19-49) | 20 (11-33) | 19 (10-30) | 18 (10-30) | 20 (11-35) | 8 (1-16) | 5 (1-13) | 1 (1-9) |
|  | Nyimanji | 30 (18-47) | 19 (10-34) | 19 (10-33) | 18 (10-32) | 18 (8-35) | 7 (1-17) | 4 (1-14) | 1 (1-8) |
|  | Olimbuni Aroga | 28 (16-46) | 20 (10-37) | 19 (9-36) | 19 (9-34) | 17 (8-32) | 8 (1-18) | 5 (1-14) | 1 (1-9) |
|  | **shift by 6 months** | | | | | | | | |
|  | Palaure Pacunaci | 35 (24-48) | 26 (16-45) | 25 (16-43) | 24 (15-41) | 27 (16-41) | 10 (1-30) | 7 (1-25) | 4 (1-19) |
|  | Masaloa | 31 (19-49) | 19 (11-33) | 19 (10-30) | 18 (10-29) | 20 (10-24) | 7 (1-20) | 5 (1-16) | 1 (1-12) |
|  | Nyimanji | 30 (17-47) | 19 (10-34) | 19 (10-33) | 18 (10-32) | 18 (8-23) | 7 (1-19) | 4 (1-15) | 1 (1-10) |
|  | Olimbuni Aroga | 28 (15-46) | 20 (10-37) | 19 (9-35) | 19 (9-34) | 16 (8-21) | 8 (1-16) | 5 (1-12) | 1 (1-7) |
| **WHO thresholds** | **shift by 1 month** | | | | | | | | |
|  | Palaure Pacunaci | 25 (15-50) | 23 (14-46) | 23 (14-40) | 22 (14-37) | 19 (9-41) | 15 (6-30) | 13 (3-25) | 9 (1-19) |
|  | Masaloa | 20 (8-35) | 19 (8-33) | 19 (8-31) | 18 (8-29) | 13 (4-24) | 10 (1-20) | 7 (1-16) | 1 (1-12) |
|  | Nyimanji | 18 (9-34) | 18 (9-31) | 17 (8-30) | 17 (8-29) | 11 (3-23) | 8 (1-19) | 5 (1-15) | 1 (1-10) |
|  | Olimbuni Aroga | 15 (3-30) | 14 (3-27) | 14 (3-26) | 13 (3-26) | 10 (1-21) | 7 (1-16) | 4 (1-12) | 1 (1-7) |
|  | **shift by 3 months** | | | | | | | | |
|  | Palaure Pacunaci | 26 (15-50) | 24 (14-50) | 23 (14-41) | 22 (14-37) | 19 (9-47) | 16 (6-32) | 13 (3-25) | 9 (1-20) |
|  | Masaloa | 20 (8-36) | 19 (8-33) | 19 (8-30) | 18 (8-29) | 13 (5-24) | 10 (2-20) | 8 (1-17) | 1 (1-12) |
|  | Nyimanji | 19 (9-34) | 18 (8-32) | 17 (8-30) | 17 (8-29) | 12 (3-24) | 9 (1-20) | 6 (1-16) | 1 (1-10) |
|  | Olimbuni Aroga | 15 (3-31) | 14 (3-28) | 14 (3-27) | 13 (3-26) | 10 (2-21) | 7 (1-17) | 4 (1-13) | 1 (1-8) |
|  | **shift by 6 months** | | | | | | | | |
|  | Palaure Pacunaci | 26 (15-50) | 24 (14-46) | 23 (14-40) | 22 (13-37) | 19 (9-42) | 16 (7-32) | 13 (2-25) | 9 (1-19) |
|  | Masaloa | 20 (8-36) | 19 (8-33) | 19 (8-30) | 18 (8-29) | 13 (5-24) | 10 (1-20) | 7 (1-17) | 1 (1-12) |
|  | Nyimanji | 19 (9-34) | 18 (8-31) | 17 (8-30) | 17 (8-29) | 11 (3-24) | 8 (1-19) | 5 (1-15) | 1 (1-10) |
|  | Olimbuni Aroga | 15 (3-30) | 14 (3-27) | 14 (3-27) | 13 (3-26) | 10 (2-22) | 7 (1-17) | 4 (1-13) | 1 (1-8) |

Supplementary Table S3. Average monthly biting rates in control and intervention sites ^1^.

| **Month** | **Average Monthly Biting Rate** | | | | | |
| --- | --- | --- | --- | --- | --- | --- |
|  | **Control Sites** | | | **Intervention Sites** | | |
|  | Okidi Center | Bajere | Elugu A1 | Pwomunu | Patille | Elegu B |
| May ‘17 | 4058 | 2693 | 3788 | 3720 | 2543 | 3908 |
| June ‘17 | 2160 | 2021 | 2816 | 975 | 49 | 79 |
| July ‘17 | 1320 | 979 | 1564 | 34 | 19 | 94 |
| August ‘17 | 143 | 101 | 195 | 86 | 49 | 165 |
| September ‘17 | 158 | 71 | 105 | 214 | 56 | 803 |
| October ‘17 | 266 | 416 | 398 | 158 | 293 | 349 |
| November ‘17 | 649 | 1166 | 484 | 416 | 480 | 818 |
| December ‘17 | 64 | 98 | 135 | 79 | 131 | 173 |
| January ‘18 | 26 | 49 | 53 | 4 | 60 | 34 |
| February ‘18 | 109 | 105 | 259 | 41 | 161 | 383 |
| March ‘18 | 109 | 686 | 720 | 274 | 191 | 904 |

Supplementary Table S4. Drug and vector control parameters

| **Parameter** | **Definition** | **Prior parameter range** | **References** |
| --- | --- | --- | --- |
| *ω* | Worm killing efficacy (instantaneous) | [0.05, 0.3] | ^2-10^ |
| *ε* | Microfilariae killing efficacy (instantaneous) | [0.95, 0.99] | ^2-5,9^ |
| _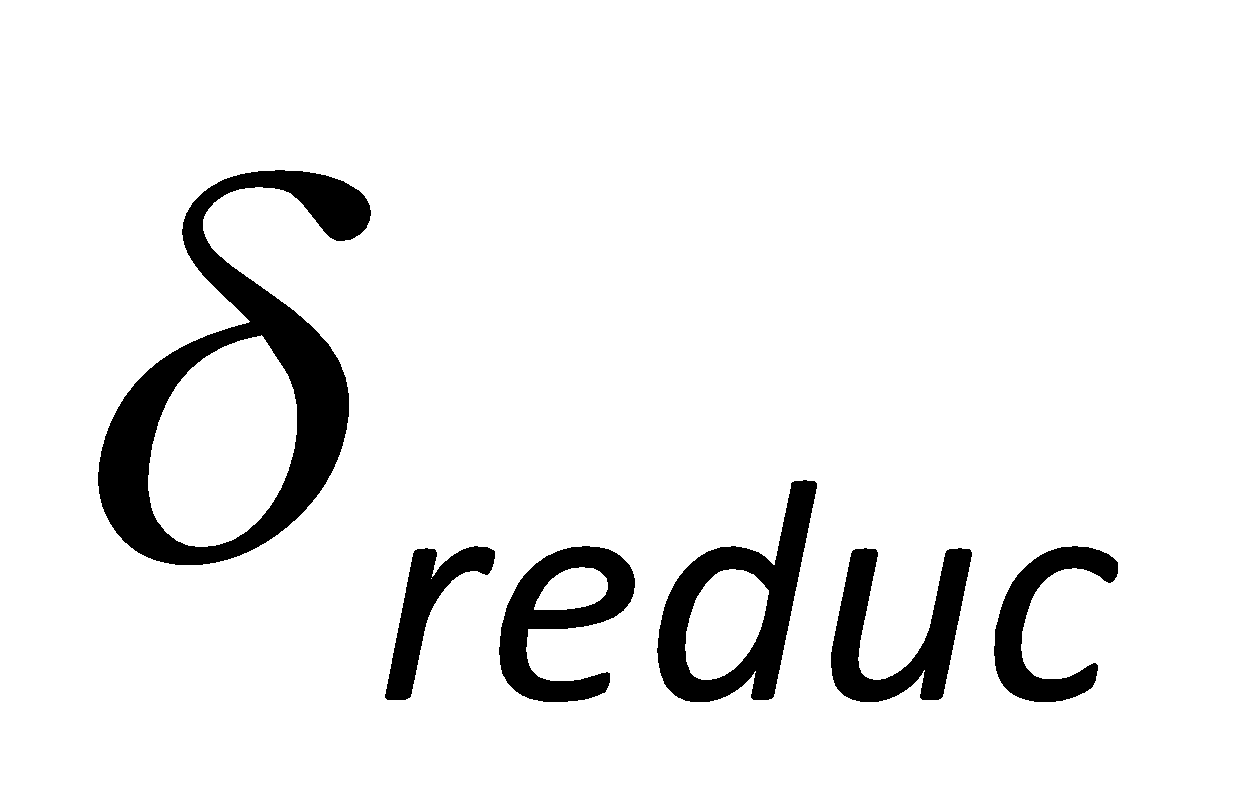_ | Reduction in microfilariae production by surviving worms | [0.05, 0.3] | ^2-5,9^ |
| *T_P_* | A time period (months) during which the drug remains efficacious in reducing the fecundity of the surviving adult worms | [9, 11] | ^3-5,7,8^ |
| *C* | Percentage of human population administered ivermectin | data | data |
| *MBR_M_* | Maximum expected monthly biting rate | data | data |
| *R_L_* | Upper rainfall threshold (mm) | [200, 400] | est. from data |
| *R_U_* | Lower rainfall threshold (mm) | [50, 250] | est. from data |
| *k_1_* | Shape parameter | [0.5, 3] | est. from data |
| *k_2_* | Shape parameter | [2, 10] | est. from data |
| *η* | Percent reduction in biting rate due to vegetation removal | [0.75, 0.95] | ^1^ |
| *Λ* | Slash and clear efficacy decay rate | [0.05, 0.4] | est. from data |

Supplementary Table S5. Description of onchocerciasis model parameters.

| **Parameter** | **Definition (units)** | **Prior parameter range** | **References** |
| --- | --- | --- | --- |
| *λ* | Number of bites per vector (per month) | = H_b_/g |  |
| *H_b_* | Human blood index | [0.3, 0.99] | ^11-15^ |
| *g* | Period of gonotrophic cycle (months) | [0.067, 0.13] | ^13,14,16,17^ |
| *V/H* | Ratio of number of vectors to hosts | MBR^1^ / *λ* | data |
| *H_Lin_^2^* | Threshold value used in *h(a)* to adjust the age-dependent exposure rate (months) | [12, 240] | ^18^ |
| *A^2^* | Coefficient describing population age distribution in *π(a)* | data | ^18^ |
| *B^2^* | Coefficient describing population age distribution in *π(a)* | data | ^18^ |
| *ψ_1_* | Proportion of L3 leaving vector per bite | [0.12, 0.7] | ^13-16,19^ |
| *ψ_2_* | Larval establishment rate^3^ | [0.02, 0.0854] | ^13-16^ |
| *c* | Strength of acquired immunity | [0.0001, 0.001] | ^20,21^ |
| *I_Cmin_* | Baseline presence of immunosuppression | [0.0025, 1] | ^20,21^ |
| *I_C_* | Strength of immunosuppression^4^ | [0.5, 5.5] | ^20,21^ |
| *S_C_* | Slope of immunosuppression function^5^ (per worm/month) | [0.1, 0.75] | ^20,21^ |
| _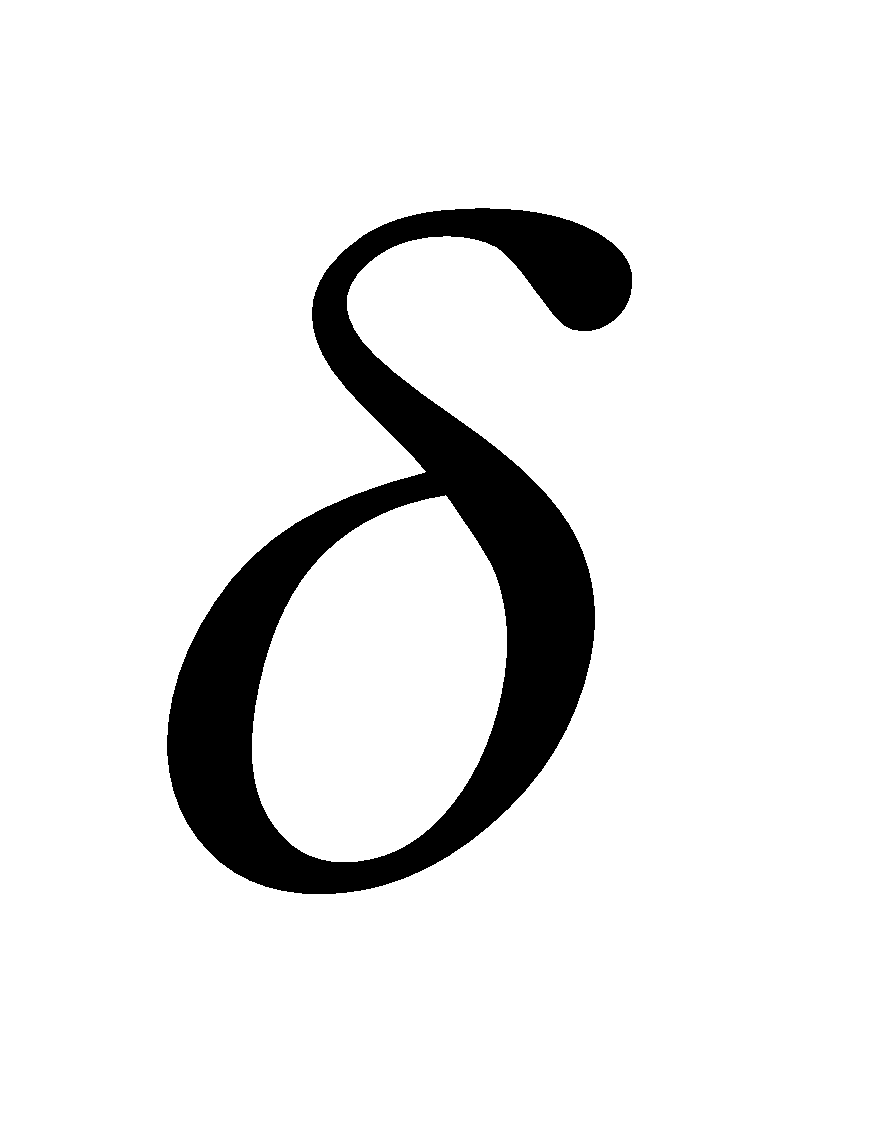_ | Immunity waning rate (per month) | [0.00001, 0.0001] | ^20,21^ |
| *μ_W_* | Worm mortality rate (per month) | [0.0083, 0.0104] | ^13-16,19^ |
| *τ* | Pre-patency period (months) | [9, 26] | ^16,19,22^ |
| *k_0_* | Basic location parameter of negative binomial distribution used in *k* | [0.00036, 0.0044] | ^20,21^ |
| *k_Lin_* | Linear rate of increase in *k* | [0.00000024, 0.282] | ^20,21^ |
| *s* | Proportion of female worms | 0.5 | - |
| *α* | Production rate of microfilariae per worm (per month) | [0.25, 1.5] | ^13-16,19^ |
| *γ* | Microfilariae mortality rate (per month) | [0.08, 0.12] | ^13-16,19^ |
| *b* | Proportion of vectors which pick up infection when biting an infected host | [0.259, 0.481] | ^13-16^ |
| *κ* | Maximum level of L3 given Mf density | [1.16, 2.00] | ^23,24^ |
| *r* | Gradient of Mf uptake^6^ | [0.01,0.0495] | ^23,24^ |
| *σ* | Vector mortality rate (per month) | [1.5, 8.5] | ^13-16^ |
| *σ_e_* | Excess vector mortality due to mf infection (per month) | [0.0208, 0.0529] | ^25^ |
| *σ_L_* | Larval mortality rate | [0.33, 1.16] | ^13-16^ |

^1^Note MBR (monthly biting rate) serves as an input to initialize the model, measured as mosquito bites per person per month, the value of which may be obtained from entomological surveys conducted in study sites. In the absence of the observed MBR value, the model has been adapted to estimate it from the community-level Mf prevalence data.

^2^The parameters *A*, *B*, and *H_Lin_* are estimated from national human demographic data or from the age-prevalence data.

^3^The proportion of L3-stage larvae infecting human hosts that survive to develop into adult worms.

^4^The facilitated establishment rate of adult worms due to parasite-induced immunosuppression in a heavily infected human host.

^5^The initial rate of increase by which the strength of immunosuppression is achieved as *W* increases from 0 ^21^.

^6^The gradient of Mf uptake *r* is a measure of the initial increase in the infective L3 larvae uptake by vector as *M* increases from 0 ^18^.

Supplementary Table S6. Description of model functions and functional forms.

| **Function** | **Functional form** |
| --- | --- |
| $h(a)$: age-dependent exposure rate | $=\left\{\begin{matrix} \frac{a}{H_{Lin}}\text{ for }a<H_{Lin} \\ 1\text{ for }a\geq H_{Lin} \end{matrix}\right.$ |
| $k$: parasite aggregation | $=k_{0}+k_{Lin}\overline{M(a,t)}$ |
| $\zeta$: rate of pre-patent worm maturation | $=e^{-\mu_{W}\tau}$ |
| :worm mating probability | $=1-\left(1+\frac{W}{2k}\right)^{-(1+k)}$ |
| $\pi(a)$: population age distribution | $=Ae^{(-Ba)}$ |
| $f[M(a,t),k]$: Vector Mf uptake response |   for vectors with cibarial armature  for vectors without cibarial armature |
| $\Phi$: larval establishment rate | $=\lambda\frac{V}{H}h(a)\psi_{1}\psi_{2}$ |
| $F_{1}\left[I\left(a,t\right)\right]$ : human immunity to larval establishment | $=\frac{1}{1+cI(a,t)}$ |
| $F_{2}\left[W_{T}(a,t)\right]$ : human immunosuppression | $=\frac{I_{C\min}+I_{C}S_{C}W_{T}(a,t)}{1+S_{C}W_{T}(a,t)}$ |
| $F_{3}$: Mf production in the human host |  |
| $F_{4}$: L3 stage larval density in the vector | $=\frac{\lambda\kappa b\int\pi(a)(1-f[M(a,t),k])da}{\sigma_{L}+\sigma+\lambda\psi_{1}+\sigma_{e}\int\pi(a)h(a)M(a,t)}$ |

## Mf age profile construction in the absence of age-stratified infection data

The Bayesian Melding procedure for calibrating our deterministic onchocerciasis model with data relies on baseline age profiles of microfilaria (mf) prevalence, but, in this study, only the overall community level mf prevalence was available for each site. This therefore required the translation of the overall prevalence into theoretical age infection profiles. This was done by firstly fitting equations to datasets (Table S6 and Table S7) of age-stratified mf infection data that qualitatively follow either plateau or convex profiles (Fig. S3) using the ‘nlme’ package in R. Specifically, plateau and convex age prevalence curves were defined by the following equations where *P* is the mf prevalence as a function of age *a*:

$$\begin{matrix} P(a)=\frac{0.29a}{1+\frac{a}{3.29}}\text{ for plateau-type age profiles, and} \\ P(a)=0.08a*\exp[-0.03a]\text{ for convex-style age profiles.} \end{matrix}$$

The number of infected individuals in each age class was then derived from the observed overall mf prevalence by applying these equations to the overall prevalence, while subdividing the total population into age classes in each site according to their respective national age-demographic patterns.

Supplementary Table S7. Onchocerciasis infection data by age for Ugandan sites exhibiting a plateau-style age profile

| **Site** | **Age** | **No. Examined** | **No. mf Positive** |
| --- | --- | --- | --- |
| Kakira | 0-10 | 13 | 6 |
|  | 11-20 | 68 | 56 |
|  | 21-30 | 69 | 59 |
|  | 31-40 | 35 | 32 |
|  | 41-50 | 25 | 25 |
|  | 51-60 | 19 | 18 |
|  | 61-70 | 7 | 7 |
| Kajuma | 0-10 | 19 | 10 |
|  | 11-20 | 60 | 48 |
|  | 21-30 | 51 | 45 |
|  | 31-40 | 49 | 48 |
|  | 41-50 | 31 | 29 |
|  | 51-60 | 7 | 6 |
|  | 61-70 | 6 | 5 |
| Nsinde | 0-10 | 5 | 3 |
|  | 11-20 | 47 | 37 |
|  | 21-30 | 12 | 9 |
|  | 31-40 | 14 | 12 |
|  | 41-50 | 10 | 8 |
|  | 51-60 | 4 | 3 |
|  | 61-70 | 98 | 76 |
| Mirambi | 0-10 | 9 | 6 |
|  | 11-20 | 69 | 59 |
|  | 21-30 | 42 | 36 |
|  | 31-40 | 35 | 30 |
|  | 41-50 | 12 | 9 |
|  | 51-60 | 11 | 10 |
|  | 61-70 | 5 | 4 |
| Buhanda | 0-10 | 9 | 5 |
|  | 11-20 | 23 | 20 |
|  | 21-30 | 13 | 11 |
|  | 61-70 | 3 | 3 |

Supplementary Table S8. Onchocerciasis infection data by age for Ugandan sites exhibiting a convex-style age profile

| **Site** | **Age** | **No. Examined** | **No. mf Positive** |
| --- | --- | --- | --- |
| Kengeyo | 0-10 | 6 | 4 |
|  | 11-20 | 27 | 24 |
|  | 21-30 | 20 | 16 |
|  | 31-40 | 3 | 2 |
|  | 41-50 | 2 | 2 |
|  | 51-60 | 1 | 1 |
|  | 61-70 | 59 | 49 |
| Kibangali | 0-10 | 9 | 6 |
|  | 11-20 | 82 | 59 |
|  | 21-30 | 61 | 56 |
|  | 31-40 | 53 | 46 |
|  | 41-50 | 39 | 35 |
|  | 51-60 | 19 | 13 |
|  | 61-70 | 13 | 10 |
| Kakasi | 0-10 | 3 | 1 |
|  | 11-20 | 9 | 8 |
|  | 21-30 | 9 | 7 |
|  | 31-40 | 3 | 3 |
|  | 41-50 | 5 | 4 |
|  | 51-60 | 5 | 3 |
|  | 61-70 | 4 | 3 |
| Ihunda | 0-10 | 6 | 2 |
|  | 11-20 | 42 | 37 |
|  | 21-30 | 18 | 16 |
|  | 31-40 | 15 | 11 |
|  | 41-50 | 5 | 4 |
|  | 51-60 | 9 | 7 |

a.


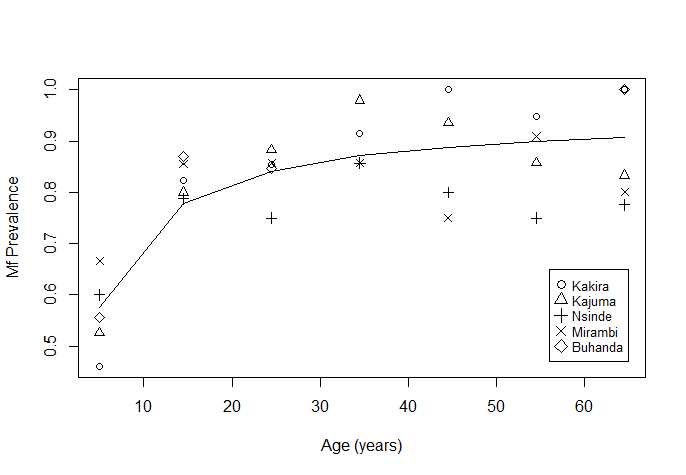

b.

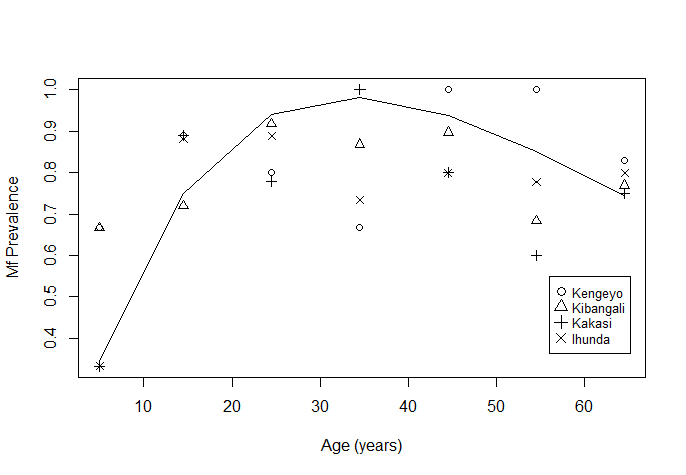


Supplementary Figure S3. NLME fits to (A) plateau- and (B) convex-style age infection profiles.

References

1 Jacob, B. G. *et al.* Community-directed vector control to supplement mass drug distribution for onchocerciasis elimination in the Madi mid-North focus of Northern Uganda. *PLoS Negl. Trop. Dis.* **12** (2018).

2 Awadzi, K., Attah, S. K., Addy, E. T., Opoku, N. O. & Quartey, B. T. The effects of high-dose ivermectin regimens on Onchocerca volvulus in onchocerciasis patients. *Trans. R. Soc. Trop. Med. Hyg.* **93**, 189-194 (1999).

3 Coffeng, L. E. *et al.* Elimination of African Onchocerciasis: Modeling the Impact of Increasing the Frequency of Ivermectin Mass Treatment. *PLoS One* **9** (2014).

4 Gardon, J., Boussinesq, M., Kamgno, J., Gardon-Wendel, N. & Duke, B. O. L. Effects of standard and high doses of ivermectin on adult worms of Onchocerca volvulus: a randomised controlled trial. *Lancet* **360**, 203-210 (2002).

5 Goa, K. L., McTavish, D. & Clissold, S. P. Ivermectin: A review of its antifilarial activity, pharmacokinetic properties and clinical efficacy in onchocerciasis. *Drugs* **42**, 640-658 (1991).

6 Habbema, J. D. F., Stolk, W. A., Veerman, L. J. & de Vlas, S. J. A rapid health impact assessment of APOC: technical report. 1-75 (2007).

7 Osei-Atweneboana, M. Y., Eng, J. K. L., Boakye, D. A., Gyapong, J. O. & Prichard, R. K. Prevalence and intensity of Onchocerca volvulus infection and efficacy of ivermectin in endemic communities in Ghana: a two-phase epidemiological study. *Lancet* **369**, 2021-2029 (2007).

8 Plaisier, A. P. *et al.* Irreversible Effects of Ivermectin on Adult Parasites in Onchocerciasis Patients in the Onchocerciasis Control Programme in West Africa. *J. Infect. Dis.* **172**, 204-210 (1995).

9 Turner, H. C., Walker, M., Churcher, T. S. & Basanez, M. G. Modelling the impact of ivermectin on River Blindness and its burden of morbidity and mortality in African Savannah: EpiOncho projections. *Parasit Vectors* **7**, 241-3305-3307-3241 (2014).

10 Walker, M. *et al.* Modelling the elimination of river blindness using long-term epidemiological and programmatic data from Mali and Senegal. *Epidemics* **18**, 4-15 (2017).

11 Garms, R. Observations on filarial infections and parous rates of anthropophilic blackflies in Guatemala, with reference to the transmission of Onchocerca volvulus. *Tropenmed. Parasitol.* **26**, 169-182 (1975).

12 Ochoa, A. Studies on the anthropophilic blackfly species in Guatemala, with special reference to the transmission of onchocerciasis in the southeastern endemic area. *Jpn J Sanit Zool* (1982).

13 Basanez, M. G. *et al.* Density-Dependent Processes in the Transmission of Human Onchocerciasis - Relationship between the Numbers of Microfilariae Ingested and Successful Larval Development in the Simuliid Vector. *Parasitology* **110**, 409-427 (1995).

14 Basanez, M.-G. & Boussinesq, M. Population biology of human onchocerciasis. *Philos. Trans. R. Soc. Lond. B Biol. Sci.* **354**, 809-826 (1999).

15 Basanez, M. G., Collins, R. C., Porter, C. H., Little, M. P. & Brandling-Bennett, D. Transmission intensity and the patterns of Onchocerca volvulus infection in human communities. *Am. J. Trop. Med. Hyg.* **67**, 669-679 (2002).

16 Filipe, J. A. *et al.* Human infection patterns and heterogeneous exposure in river blindness. *Proc. Natl. Acad. Sci. U. S. A.* **102**, 15265-15270 (2005).

17 Basanez, M.-G., Razali, K., Renz, A. & Kelly, D. Density-dependent host choice by disease vectors: epidemiological implications of the ideal free distribution. *Trans. R. Soc. Trop. Med. Hyg.* **101**, 256-269 (2007).

18 Norman, R. A. *et al.* EPIFIL: the development of an age-structured model for describing the transmission dynamics and control of lymphatic filariasis. *Epidemiol. Infect.* **124**, 529-541 (2000).

19 Duke, B. O. Observations and reflections on the immature stages of Onchocerca volvulus in the human host. *Ann. Trop. Med. Parasitol.* **85**, 103-110 (1991).

20 Duerr, H. P., Dietz, K., Schulz-Key, H., Büttner, D. W. & Eichner, M. Density-dependent parasite establishment suggests infection-associated immunosuppression as an important mechanism for parasite density regulation in onchocerciasis. *Trans. R. Soc. Trop. Med. Hyg.* **97**, 242-250 (2003).

21 Duerr, H.-P., Dietz, K. & Eichner, M. Determinants of the eradicability of filarial infections: a conceptual approach. *Trends Parasitol* **21**, 88-96 (2005).

22 Duerr, H. P. & Eichner, M. Epidemiology and control of onchocerciasis: the threshold biting rate of savannah onchocerciasis in Africa. *Int. J. Parasitol.* **40**, 641-650 (2010).

23 Grillet, M.-E. *et al.* Vector competence of Simulium oyapockense and S. incrustatum for Onchocerca volvulus: Implications for ivermectin-based control in the Amazonian focus of human onchocerciasis, a multi-vector–host system. *Acta Trop.* **107**, 80-89 (2008).

24 Soumbey-Alley, E. *et al.* Uptake of Onchocerca volvulus (Nematoda: Onchocercidae) by Simulium (Diptera: Simuliidae) is not strongly dependent on the density of skin microfilariae in the human host. *J. Med. Entomol.* **41**, 83-94 (2004).

25 Basáñez, M. G. *et al.* Density-dependent processes in the transmission of human onchocerciasis: relationship between microfilarial intake and mortality of the simuliid vector. *Parasitology* **113**, 331-355 (1996).
